# Creutzfeldt–Jacob disease: new directions in diagnosis and therapeutics

**DOI:** 10.1007/s00415-017-8473-4

**Published:** 2017-03-28

**Authors:** Aseel Al-Ansari, N. P. Robertson

**Affiliations:** Institute of Psychological Medicine and Clinical Neurosciences, Cardiff University, Cardiff, CF14 4XW UK

Creutzfeldt–Jacob disease (CJD) is a rapidly progressive neurodegenerative disorder characterised by the conformational change of prion protein into an abnormal form which then self-propagates and deposits in the brain. Diagnosis is currently based on a combination of the clinical picture, MRI and EEG findings together with the detection of protein 14-3-3 in CSF. However, many of the traditional diagnostic features are non-specific and only manifest later in the disease course. As a result, an early confident diagnosis of CJD is often problematic, which may in turn disadvantage any time-dependent potential therapies.

This month, we discuss three papers which assess new approaches to the diagnosis and treatment of CJD. The first paper publishes the results of an international study examining the reliability of real-time quaking-induced conversion (RT-QuIC) as a diagnostic test for CJD. The second paper is a cross-sectional study assessing the ability of an assay to diagnose sporadic CJD by the detection of abnormal prion protein in patient urine. The third paper is a double-blinded randomised phase II and observational study looking at the therapeutic effect of Doxycycline in sporadic CJD.

## Cerebrospinal fluid real-time quaking-induced conversion is a robust and reliable test for sporadic Creutzfeldt–Jacob disease: an international study

RT-QuIC has previously been shown to be a sensitive and specific diagnostic test for sporadic CJD. This technique uses a recombinant prion protein as a substrate, which aggregates once disease-causing prion protein is added. This study set out to confirm the reliability of RT-QuIC as a diagnostic test by demonstrating its reproducibility across different international centres using a range of recombinant prion protein substrates and instrumentation.

The study comprised two international ring trials. The initial ring comprised seven European participants; the second ring comprised 11 laboratories to include participants from Australia, Canada and Japan. CSF was provided by the National CJD Unit, United Kingdom and identical samples were analysed blind by each of the participating laboratories. Each laboratory used a standard buffer solution, however, a range of instrumentation, analytical conditions and types of recombinant prion protein were used.

In the first ring trial, seven European laboratories received ten identical CSF samples from ten patients. Five samples were from patients who had confirmed or probable sCJD with disease duration of between 2 and 12 months and five samples came from patients with other neurological pathology. Six of seven laboratories obtained positive RT-QuIC responses from all five CJD cases, one laboratory obtained positive RT-QuIC responses from only four of the five CJD cases. None of the laboratories obtained positive RT-QuIC results in any of the five samples from patients with alternative neurological pathology. In the second ring trial, 15 CSF samples were sent to 11 centres. Eight patients had confirmed or probable CJD with disease duration of between 1 and 26 months. The remaining seven had other neurological pathology. All 11 laboratories correctly identified the eight CSF samples from CJD patients and none detected a positive RT-QuIC result in CSF from patients without CJD. The study concludes that an overall sensitivity of between 85.7 and 100% and specificity of 100% was achieved in the utility of RT-QuIC in detecting sporadic CJD.

*Comment*. Although the numbers of patients in both trials are small, the sensitivity and specificity values demonstrate the reproducibility of this technique across international laboratories despite the use of different recombinant protein, instrumentation and analytic conditions. The wide variation in the age range and duration of disease of the patients with CJD in both ring trials implies that this technique is capable of picking up sporadic CJD in early as well as later stages. It should be noted that only sporadic CJD patients are included in this study; no information is provided on the utility of RT-QuIC in diagnosing variant, inherited or iatrogenic CJD.

McGuire et al (2016) Ann Neurol 80:160–165

## Diagnosing sporadic Creutzfeldt–Jacob disease by the detection of abnormal prion protein in patient urine

Non-invasive detection of the abnormal prion protein would clearly be a significant advance in the management of CJD. Previous assays have detected abnormal protein in blood and urine of variant but not sporadic CJD. This cross-sectional, retrospective study assesses the ability of an assay, developed from a blood test for vCJD, to detect abnormal protein in the urine of patients with sporadic CJD. The assay captures disease-associated PrP on a stainless steel matrix detected using anti-PrP monoclonal antibodies.

A total of 162 samples were analysed: 91 healthy control individuals, 34 patients with non-prion degenerative diseases and 37 patients with prion disease (20 of whom had sporadic CJD). Each sample was tested using triplicate wells with a quality control panel on each plate and tested across two independent runs. An arbitrary cut off was determined from the mean plus five SDs of the panel of five normal samples in the quality control panel. Only samples that were reactive in both test runs were scored as positive. In 55 samples conductivity of the urine samples was also measured; no correlation was identified between urine concentration and direct detection assay signals, indicating patient hydration was not responsible for elevated PrP signals.

Of the 162 blinded samples, ten samples scored positive across both runs. All ten positive samples were from patients with prion disease, of which eight were from patients with sporadic CJD. No clear correlation was found with aspects of clinical history, disease progression or investigation results. The authors concluded that the assay detected 27% of CJD and 40% of sCJD samples.

*Comment.* This is the first study to demonstrate detection of abnormal prion protein in urine in sCJD. Although the sensitivities demonstrated are low, the authors point out that the high mean signal obtained for sCJD samples suggests that the sensitivity could be improved considerably by pre-treatment of urine samples. This study uses good numbers of patients and sound methodology involving triplicate analysis of urine samples as well as two independent runs. However, there may be difficulties in standardising and reproducing methodology in different laboratories.

Luk C et al (2016) JAMA Neurol 72(12):1454–1460

## Doxycycline in early CJD: a double-blinded randomised phase II and observational study

A number of drugs have been tried and failed in clinical trials of CJD. Doxycycline has been reported to act as an anti-prion agent in both in vitro and animal models of CJD, however, a recent phase II trial failed to demonstrate efficacy. It has subsequently been postulated that this may have been the result of inclusion of patients mainly in advanced stage of disease. Authors of this study therefore aimed to explore this further by exploring the therapeutic efficiency of Doxycycline in the early stages of CJD.

Two groups of patients were enrolled. The first group was recruited from the National Reference Centre of TSE surveillance in Germany. Patients with probable or definite CJD were classified according to established disease criteria. Date of disease onset was determined by interview with relatives and only patients with a disease duration of less than 24 months were included. Additional inclusion criteria were: MMSE >6 and absence of known PRNP mutations. Seven patients were randomly assigned to the treatment arm with Doxycycline 100 mg daily and five to the placebo arm. Disease progression was evaluated by standardised questionnaire. The second group comprised patients treated compassionately with Doxycycline. At the time of diagnosis patients with sCJD were seen by a physician and assessed with special emphasis on CJD specific symptoms and MMSE. Treatment with Doxycycline (100 mg daily) was initiated and patients followed up per protocol. Seventy-seven patients were included although 22 excluded as a result of an unknown baseline MMSE, leaving 55 patients for analysis. A matched, treatment-naive control group of 33 patients with confirmed or probable sCJD was identified from historical data. The primary outcome measure was survival time, with quality of life as a secondary outcome measure.

In the randomised, double-blinded placebo controlled trial, no statistically significant prolongation of survival time or improvement in quality of life was observed. However, in the observational study, survival time was increased compared to controls which the authors postulate may be a genotype-specific effect since the results suggested this was most apparent in patients MM homozygous at codon 129. The results of both studies were formally combined in a meta-analysis demonstrating statistically significant superiority (*p* = 0.049) of Doxycycline treatment over control.

*Comment.* The double-blinded randomised controlled trial has small numbers which may be a reflection of the fact that patients and their families do not wish to be randomised to placebo versus a potentially life-prolonging treatment. Furthermore, the groups in the randomised control trial appear poorly matched—with the median age in the control group being 72 years and the Doxycycline group being 58 years. Furthermore, analysis of survival from onset, whilst clearly of value, is likely to be difficult to quantify accurately as the result of recall bias and other factors. In addition, variation between individual progression from onset of disease to diagnosis will ultimately culminate in lead time bias, which is difficult to account for. The observational study had larger patient numbers but it remains unclear how study methodology was adapted to ensure the patients were enrolled at an earlier stage of disease. Finally, the duration of treatment with Doxycycline was not made clear. The authors also noted that the cohort demonstrates an atypical distribution of codon 129 genotypes which they concede may be a result of their selection criteria.

Varges D, Manthey H, Heinemann U et al (2017) J Neurol Neurosurg Psychiatry 88:119–125

## Conclusion

The results of these studies demonstrate some encouraging progress in the development of new diagnostic and therapeutic techniques in Creutzfeldt–Jacob disease. Real-time induced quaking is already being introduced into laboratories as a reliable method of detecting sporadic CJD and there is hope that less invasive tests for CJD using patient urine and blood may be on the horizon. However, treatment of CJD remains problematic and the search for effective therapeutic agents continues.

